# Evaluation of Uric Acid to Albumin Ratio as a Marker of Coronary Artery Disease Severity in Acute Coronary Syndrome: A Cross-Sectional Study

**DOI:** 10.7759/cureus.49454

**Published:** 2023-11-26

**Authors:** Sana Sultana, Mohammed Suhail K, Varsha Rakshitha Prakash, Aditya Karthikeyan, Shaikh Mohammed Aslam S, Suhas G C, Ashwin Kulkarni

**Affiliations:** 1 General Medicine, Ramaiah Medical College & Hospital, Bangalore, IND; 2 Cardiology, Ramaiah Medical College & Hospital, Bangalore, IND

**Keywords:** uric acid albumin ratio, albumin, uric acid, syntax score, coronary artery disease

## Abstract

Background: Coronary artery disease (CAD) is a widespread cause of morbidity and mortality. Serum uric acid, a mediator of endothelial dysfunction and inflammation in vascular disease, can increase the risk of atherosclerosis, contributing to CAD. As serum albumin inhibits platelet activation and aggregation, low levels of it can contribute to platelet-induced coronary artery stenosis. Limited studies have been conducted worldwide in evaluating the role of uric acid to albumin ratio (UAR) in predicting severity or poor outcomes in acute coronary syndrome (ACS) patients. This study was undertaken to assess the role of UAR as a predictor of CAD severity, which can facilitate the identification of high-risk patients.

Methodology: A hospital-based analytical cross-sectional study was conducted in an urban tertiary healthcare center for a period of two months between June and August of 2022. A total of 100 ACS patients were included in the study. The study population included patients above the age of 18 years diagnosed with ACS who underwent a coronary angiography. Coronary angiograms were used to diagnose the presence of CAD, and its severity was assessed using Synergy between Percutaneous Coronary Intervention with Taxus and Cardiac Surgery (SYNTAX) scores (SS). The correlation of UAR with CAD severity using SS was studied and compared between three varieties of ACS: ST-elevation myocardial infarction (STEMI), non-ST-elevation myocardial infarction (NSTEMI), and unstable angina (UA).

Statistics: Chi-squared tests were used to determine statistical significance for qualitative data. Independent t-tests were used to identify the mean difference between two quantitative variables. Receiver operating characteristic (ROC) curves were constructed for UAR and high SS. A comparison between UAR and neutrophil to lymphocyte ratio (NLR) as a predictor of disease severity was done. ROC and optimal cutoff points were chosen to calculate sensitivity, specificity, and positive and negative predictive values. Microsoft Excel (Microsoft, Redmond, WA, USA) and SPSS V22.0 (IBM Corp., Armonk, NY, USA) were used to analyze the data.

Results: A total of 100 ACS patients were included in the study and divided into two groups on the basis of SS, with 74% showing low severity and 26% showing intermediate-high severity. There was a statistically significant difference found between older age and SS (p=0.017). Our study showed 74% (n=74) of the patients were male and 26% (n=26) were female. It also revealed that 75.7% (n=56) of the male patients were in the low-severity group, and 24.3% (n=18) of males were in the intermediate-high severity group. 69.2% (n=18) of the female patients were in the low-severity group, and 30.8% (n=8) were in the intermediate-high severity group.

Of the 100 patients, 55% were diagnosed with STEMI, of which 69.1% were in the low-severity group, and 30.9% were in the intermediate-high severity group. Among all the patients 33% of the patients were diagnosed as NSTEMI, of which 72.7% were in the low-severity group, and 27.3% were in the intermediate-high severity group. Twelve percent of the patients were diagnosed with UA, and 100% of these patients were in the low-severity group. The mean UAR was 1.40 ± 0.38 in the low-severity group and 1.29 ± 0.46 in the intermediate-high severity group (p=0.22).

Conclusion: Our study yielded no statistically significant difference in UAR among varying severities of CAD.

## Introduction

Cardiovascular diseases (CVDs) have now become the leading cause of mortality in India. A quarter of all mortality is attributable to CVD. Ischemic heart disease and stroke are the predominant causes and are responsible for >80% of CVD deaths. Acute coronary syndrome (ACS) is an ischemic heart disease that comprises three entities: ST-elevation myocardial infarction (STEMI), non-ST-elevation myocardial infarction (NSTEMI), and unstable angina (UA) [1].

The pathogenesis of ACS involves changes in acute atherosclerotic plaque such as rupturing, fissuring, or ulceration, which can lead to the formation of an occlusive thrombus with platelet adhesion and aggregation. Serum uric acid, a mediator of endothelial dysfunction and inflammation in vascular disease [2], can increase the risk of atherosclerosis, contributing to the occurrence of CAD as well as influencing its prognosis [3]. Serum albumin inhibits platelet activation and aggregation, and low levels of it can contribute to platelet-induced coronary artery stenosis [4,5]. Low albumin independently has been linked to worse outcomes following acute coronary syndrome and with increased likelihood of complications. Also it has been associated with increased development of complications including dementia in atrial fibrillation patients. Low levels of serum albumin can also increase blood viscosity and disrupt endothelial function [6-10].

According to recent studies, the normal range was 3.5-7.2 mg/L for uric acid and 35-52 g/L for serum albumin and the ratio of the aforementioned contributory laboratory parameters, namely, the uric acid to albumin ratio (UAR), is an independent predictor of severity of CAD in NSTEMI, and mortality in STEMI and UA patients hence these studies have described UAR as an independent predictor of severity in ACS, with elevated values associated with worse outcomes [11-13]. Further studies noted implications of elevated UAR in increased risk of new-onset atrial fibrillation, contrast-induced nephropathy, and no-reflow following percutaneous coronary intervention [14-16].

In the aforementioned studies, UAR was calculated in ACS patients by dividing serum uric acid level (mg/dL) by serum albumin level (g/dL), which was compared to CAD severity, as measured by the anatomic Synergy between Percutaneous Coronary Intervention with Taxus and Cardiac Surgery (SYNTAX) score (SS). The SS was calculated on the basis of significant lesions (>50% stenosis) on the coronary angiogram (as per SYNTAX scoring definitions). The lesions were scored, using the SS, for total occlusion, bifurcation or trifurcation lesions, severe tortuosity, heavy calcification, aorto-ostial location, thrombus burden, and diffuse disease, and patients were divided into low (<22) and intermediate-high severity (>22) groups.

Although the role of UAR in assessing ACS severity on the basis of SS has been studied exclusively in NSTEMI patients, there are no available data regarding the predictive value of UAR in ACS as a whole. There is also a lack of information on its predictive value in UA, NSTEMI, and STEMI. Furthermore, no studies have been conducted in India evaluating the role of UAR in predicting severity or poor outcomes in ACS patients.

Therefore, we conducted a study in patients with ACS to evaluate if UAR could be used as an additional tool to identify high-risk ACS patients.

## Materials and methods

A hospital-based analytical cross-sectional study was conducted in an urban tertiary healthcare center for a period of two months between June and August of 2022. A total of 100 ACS patients were included in the study.

The study population included adults >18 years of age diagnosed with ACS who underwent a coronary angiography. Demographic details such as age and gender, and clinical history for cardiovascular risk factors such as hypertension, diabetes mellitus, smoking, or previous history of CAD were noted from the case records. Patients were excluded from the study if they met the following criteria: previous history of CAD treated with percutaneous transluminal coronary angioplasty or coronary artery bypass graft, chronic liver disease, chronic kidney disease, autoimmune disease, malignancy, active infection, chronic inflammatory disease, or pregnancy.

Diagnosis of ACS was based on American College of Cardiology(ACC)/American Heart Association guidelines using symptoms, examination findings, electrocardiogram (ECG) features, and cardiac biomarkers troponin I and creatine kinase-MB (CK-MB). This is the universal definition of ACS which is endorsed by ACC amongst many others. Patients were characterized as STEMI, NSTEMI, or UA on the basis of the ECG and cardiac biomarkers. Patients with elevated cardiac biomarkers and ST elevation ECG changes were categorized as STEMI. Patients with an absence of ST elevation changes but the presence of other significant ECG changes with elevated cardiac biomarkers were categorized as NSTEMI. Patients with presence of angina without elevated cardiac biomarkers were categorized as UA.

Coronary angiograms were used to determine the presence of CAD, and anatomic severity was determined using SS, which was calculated on the software downloaded from http://www.syntaxscore.com.

Significant lesions were further assessed for total number of lesions, the presence of total occlusion, whether the lesion was a bifurcation or trifurcation lesion, and lesion site (e.g., aorto-ostial lesion). Furthermore, each chronic total occlusion was scored based on the chronicity of the lesion, the presence of blunt stumps, the presence of bridging collaterals, first segment beyond the occlusion visualized by antegrade or retrograde filling, and side branch involvement.

The study population was divided into two groups based on CAD severity SS: ≤ 22 for low severity and > 22 for intermediate-high severity. Left ventricular ejection fraction (LVEF) was noted using the modified Simpson method in apical four-chamber and two-chamber views in both end diastole and end systole [17].

Laboratory parameters

Blood samples drawn from the median cubital vein, cephalic vein, or veins on the dorsum of the hand were used for laboratory measurements before reperfusion or heparin therapy. Serum uric acid and serum albumin were measured using Roche cobasc50/e601 automated analyzer. The normal range of serum uric acid was taken as 3.4-7 mg/dL, and that of serum albumin was taken as 3.5-5.2 g/dL.

UAR was obtained by dividing the serum uric acid level (mg/dL) by the serum albumin level (g/dL). Likewise, the neutrophil to lymphocyte ratio (NLR) was calculated by dividing the neutrophil count in cells/mm3 by the lymphocyte count in cells/mm3.

Correlation of UAR with the severity of the CAD was studied and compared between the three varieties of ACS. Neutrophil and lymphocyte values were also noted from complete blood count testing. UAR and NLR were also compared as markers of CAD severity.

Statistical methods

The demographic and clinical data were entered into Microsoft Excel (Microsoft, Redmond, WA, USA) and analyzed using SPSS V22.0 (IBM Corp., Armonk, NY, USA) software. Categorical data are represented as frequencies and proportions. Chi-squared tests were used to determine statistical significance for qualitative data. Continuous data are represented as means and standard deviations. Independent Student’s t-tests were used to determine statistical significance between quantitative variables.

Receiver operating characteristic (ROC) curves were constructed for UAR and SS. A comparison was done between the classified UAR values and NLR to assess if NLR could be used as a tool to identify high-risk ACS patients. ROC and optimal cutoff points were chosen to calculate sensitivity, specificity, and positive and negative predictive values of UAR and NLR. As a test that predicts an outcome no better than chance has an area under the ROC curve of 0.5, an area under the ROC curve above 0.8 indicated fairly good prediction ability in this study.

Microsoft Excel and Word were used to obtain various types of graphs, such as bar diagrams and pie diagrams. In this study, a p-value of <0.05 was considered statistically significant.

## Results

A total of 100 ACS patients were included in the study, who were divided into two groups according to SS: a low-severity group (n=74, 74%) and an intermediate-high severity group (n=26, 26%).

**Figure 1:**
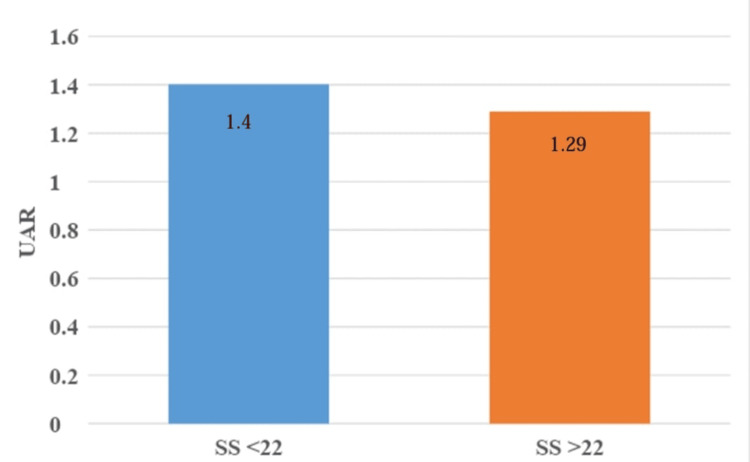
Graph showing Comparison of mean uric acid to albumin ratio according to SYNTAX score (SS).

**Figure 2:**
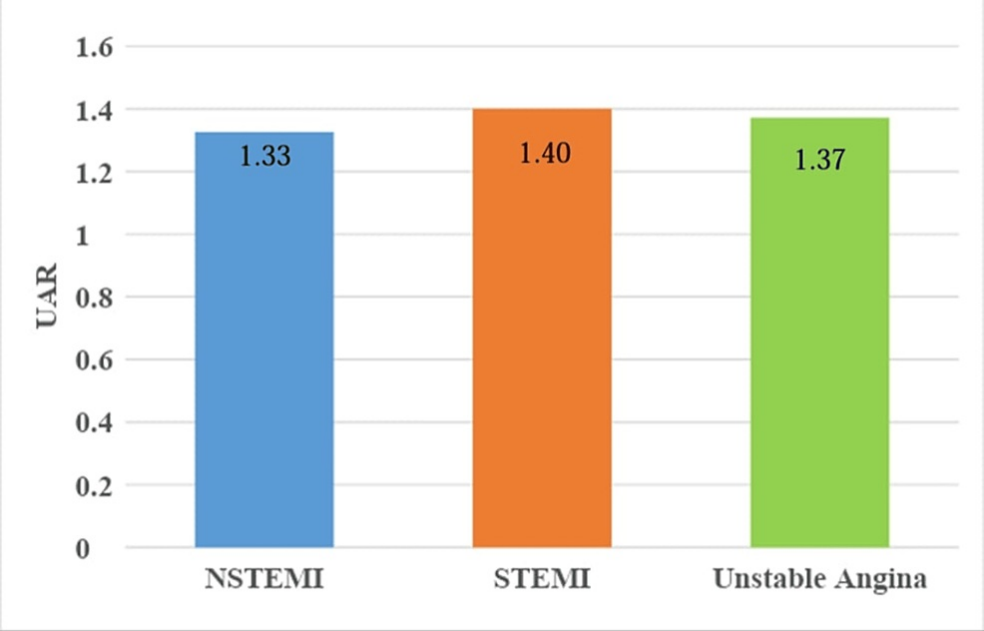
Graph showing comparison of mean uric acid to albumin ratio (UAR) to diagnosis of the type of acute coronary syndrome. STEMI = ST elevation myocardial ischemia, NSTEMI = non-ST elevation myocardial ischemia

The mean age of all patients was 61.43 years, that of the low-severity group was 59.64 years, and that of the intermediate-high severity group was 66.54 years. There was a statistically significant difference found between increased age and high SS (p=0.017; Table 1, Figure 1). Seventy-four percent of the patients were male (n=74), and 26% (n=26) were female. 75.7% (n=56) of the male patients were in the low-severity group, and 24.3% (n=18) were in the intermediate-high severity group. 69.2% (n=18) of the female patients were in the low-severity group, and 30.8% (n=8) were in the intermediate-high severity group (Table 2, Figure 2).

**Table 1:** Comparison of mean age according to SYNTAX score (SS)

SS	Mean Age (Years)	Std. Deviation	P Value
SS <22	59.64	13.262	0.017
SS>22	66.54	9.949	

**Table 2:** Distribution of subjects according to sex and SYNTAX score (SS).

Gender	SS <22		SS >22	
	N	%	N	%
Female	18	69.2%	8	30.8%
Male	56	75.7%	18	24.3%

Of the 100 patients, 55% were diagnosed with STEMI, with 69.1% of these patients in the low-severity group, and 30.9% in the intermediate-high severity group. Thirty-three percent of the patients were diagnosed with NSTEMI, with 72.7% of these patients in the low-severity group and 27.3% in the intermediate-high severity group. Twelve percent of the patients were diagnosed with UA, with 100% of these patients in the low-severity group (Table 3).

**Table 3:** Distribution of subjects according to diagnosis and SYNTAX score (SS). STEMI = ST elevation myocardial ischemia, NSTEMI = non-ST elevation myocardial ischemia

Diagnosis	SS < 22		SS >22	
	N	%	N	%
STEMI	38	69.1%	17	30.9%
NSTEMI	24	72.7%	9	27.3%
Unstable Angina	12	100.0%	0	0%

Forty percent of the patients were hypertensive, 51% were diabetic, and 10% had a history of smoking (Table 4). The mean LVEF was 46.38% for all patients, 47.2% for the low-severity group, and 43.42% for the intermediate-high severity group. There was a statistically significant difference found between LVEF and SS (p=0.005; Table 5).

**Table 4:** Distribution of subjects according to comorbidities and SYNTAX score (SS).

Comorbidities	SS <22		SS >22		P value
	N	%	N	%	
Hypertension	26	65.0%	14	35.0%	0.108
Diabetes	31	60.8%	20	39.2%	0.003
Smoking history	10	100.0%	0	.0%	0.060

**Table 5:** Comparison of mean left ventricular ejection fraction (LVEF) according to SYNTAX score (SS).

SS	Mean LVEF	Std. Deviation	P Value
SS <22	47.42	5.544	0.005
SS >22	43.42	7.398	

The mean serum uric acid level was 5.49 mg/dL for all patients, 5.65±1.42 in the low-severity group, and 5.04±1.33 in the intermediate-high severity group. There was no statistically significant difference found between serum uric acid level and SS (p=0.058; Table 6). The mean serum albumin level was 4.06 g/dL for all patients, 4.07±0.44 g/dL in the low-severity group, and 4.01±0.47 g/dL in the intermediate-high severity group. There was no statistically significant difference found between serum albumin level and SS (p=0.548; Table 7).

**Table 6:** Comparison of mean uric acid levels according to SYNTAX score (SS).

SS	Mean Uric Acid Level	Std. Deviation	P Value
SS <22	5.65	1.42	0.058
SS >22	5.04	1.33	

**Table 7:** Comparison of mean serum albumin levels according to SYNTAX score (SS).

SS	Mean Serum Albumin Level	Std. Deviation	P Value
SS <22	4.07	0.44	0.548
SS >22	4.01	0.47	

**Figure 3:**
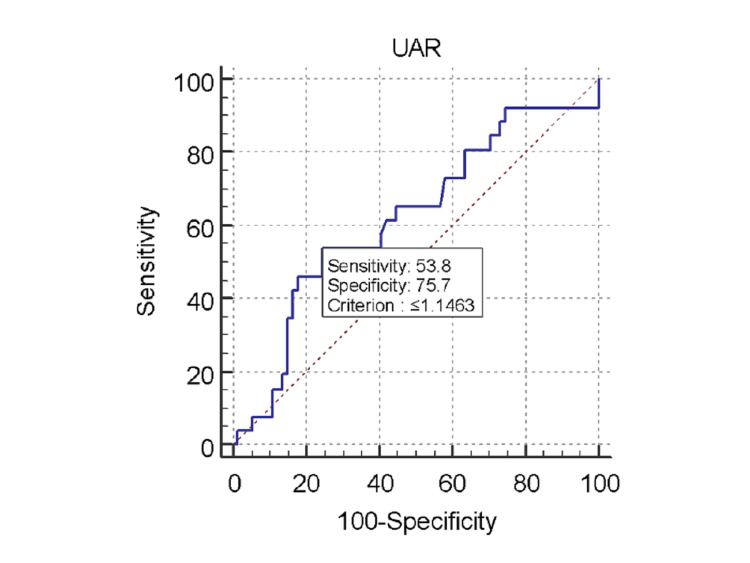
Receiver operating characteristic curve for uric acid to albumin ratio (UAR) in predicting high SYNTAX score.

The mean UAR was 1.40±0.38 in the low-severity group and 1.29±0.46 in the intermediate-high severity group. There was no statistically significant association found between UAR and SS (p=0.22; Figure 1). The mean UAR was 1.4006±0.48 for the STEMI patients, 1.326±0.3077 for the NSTEMI patients, and 1.372±0.219 for the UA patients. There was no statistically significant difference found between type of ACS diagnosed and UAR (Figure 2). By ROC assessment, the area under the curve (AUC) threshold for UAR to predict high SS was 0.621, with a specificity of 75.7%, a sensitivity of 53.8%, and a cutoff value of <1.146 (Table 8, Figure 3).

**Table 8:** Sensitivity, specificity, negative predictive value (NPV) and positive predictive value (PPV) for uric acid to albumin ratio predicting high SYNTAX score.

Cut off	Sensitivity	95% CI	Specificity	95% CI	+PV	95% CI	-PV	95% CI
≤1.1463	53.85	33.4 - 73.4	75.68	64.3 - 84.9	43.8	26.4 - 62.3	82.4	71.2 - 90.5

**Figure 4:**
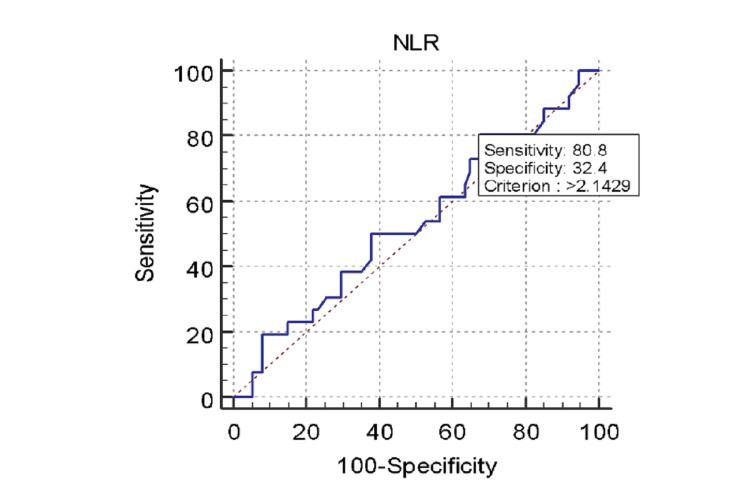
Receiver operating characteristic curve for neutrophil to lymphocyte ratio (NLR) in predicting high SYNTAX score.

**Figure 5:**
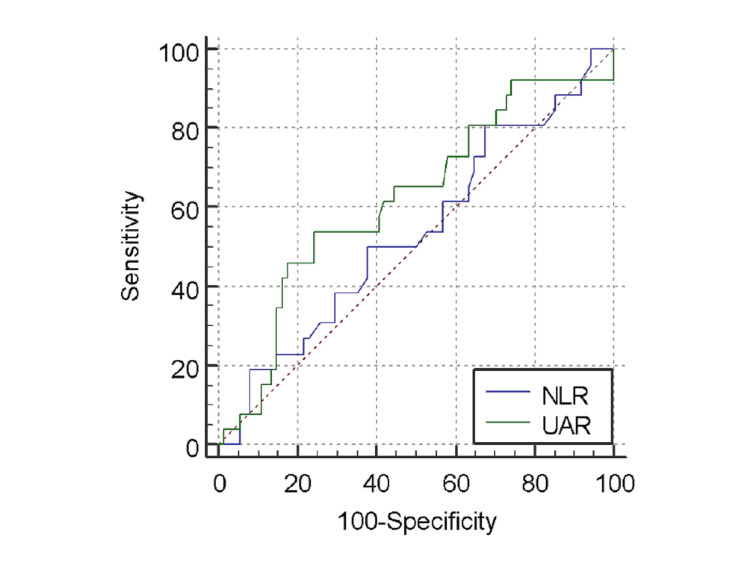
Comparison of receiver operating characteristic curves for neutrophil to lymphocyte ratio (NLR) and uric acid to albumin ratio (UAR) in predicting high SYNTAX score.

There was no significant difference between total leukocyte count, neutrophil%, or lymphocyte% between the low and intermediate-high severity groups (Table 9). The mean NLR was 4.3625. There was no statistically significant difference found between NLR and SS (Table 10). The mean NLR was 4.4327±3.4626 in the STEMI patients, 4.3756±4.9968 in the NSTEMI patients, and 4.0046±4.8181 in the UA patients (Table 11). There was no statistically significant difference found between diagnosis of ACS type and NLR (p=0.950) (Figure 4). According to the ROC assessment, the AUC threshold for NLR to predict high SS was 0.535, with a specificity of 32.4%, a sensitivity of 80.8%, and a cutoff value of >2.1429 (Table 12, Figure 5). UAR had a better AUC than NLR and was, therefore, better at predicting high SS.

**Table 9:** Comparison of total leukocyte count, neutrophil%, and lymphocyte% between the low and intermediate-high SYNTAX score (SS) groups.

Metric	SS <22		SS >22		
	Mean	SD	Mean	SD	P value
Total Leukocyte Count	11040.27	3339.59	10666.92	3102.76	0.691
Neutrophils %	67.03	12.81	69.27	11.81	0.436
Lymphocytes %	23.24	10.71	22.15	10.65	0.656

**Table 10:** Comparison of mean neutrophil to lymphocyte ratio according to SYNTAX score (SS).

SS	Mean ( Neutrophil to lymphocyte ratio )	Std. Deviation	p value
SS <22	4.326163	4.3834498	0.883
SS >22	4.465889	3.4671416	

**Table 11:** Comparison of mean neutrophil to lymphocyte ratio according to acute coronary syndrome diagnosis. STEMI = ST elevation myocardial ischemia, NSTEMI = non-ST elevation myocardial ischemia

Diagnosis	NLR	
Acute Coronary Syndromes (Types)	Mean	SD
STEMI	4.4327	3.4626
NSTEMI	4.3756	4.9968
Unstable Angina	4.0046	4.8181

**Table 12:** Sensitivity, specificity, negative predictive value (NPV) and positive predictive value (PPV) for neutrophil to lymphocyte ratio (NLR) in predicting high SYNTAX score.

Cut off	Sensitivity	95% CI	Specificity	95% CI	+PV	95% CI	-PV	95% CI
>2.1429	80.77	60.6 - 93.4	32.43	22.0 - 44.3	29.6	19.3 - 41.6	82.8	64.2 - 94.2

## Discussion

The above study was conducted to determine the role of UAR as a marker of the anatomic severity of CAD in patients diagnosed with ACS. Based on our results, increased UAR was not significantly associated with a higher SS and therefore could not predict the anatomic severity of CAD in ACS. There was no significant difference between UAR in STEMI, NSTEMI, and UA patients. In contrast, a similar retrospective study by Cakmak et al. [11] found statistical significance between UAR and CAD severity in NSTEMI patients.

Serum uric acid and serum albumin levels also did not show significant correlations with SS, whereas according to Cakmak et al. [11], the former was higher and the latter was lower in the intermediate-high severity group. This difference can most likely be explained by the relatively smaller sample size in our study and the inclusion of patients with minor CAD as diagnosed by coronary angiogram without significant lesions, which had an SS of 0.

NLR was not associated with disease severity and did not correlate with SS in the ACS patients. Similarly, in a study by Cakmak et al. [11], NLR was also not found to be statistically significant in determining CAD severity in NSTEMI patients.

However, a meta-analysis of 17 studies suggested that high NLR was associated with severity of CAD and could be useful for predicting severe stenosis [18,19].

In one of the studies done by Xing et al. [20], C-reactive protein (CRP) predicts high clinical SYNTAX score (CSS) at a lower level than it predicts SYNTAX score (SXscore). Thus, serum CRP combined with serum uric acid (UA) may be useful to predict SXscore and CSS.

According to our study, reduced LVEF was significantly associated with high SS, which was in accordance with the study by Cakmak et al. [11]. However, unlike the Turkish study, we also found that increased age was associated with high SS [11].

To the best of our knowledge, this is the first study evaluating the role of UAR in the prediction of CAD severity in ACS as a whole and its significance in STEMI, NSTEMI, and UA patients. This is also the first study evaluating the predictive role of UAR in CAD conducted in an Indian setting. Our study did have its limitations. First, our study had a relatively small sample size of 100 patients compared to similar studies. Second, the laboratory parameters were measured only on admission without serial follow-up.

## Conclusions

Our study yielded no statistically significant differences in UAR among varying severities of CAD as evaluated by SS in ACS patients in an Indian population. There was no correlation found between UAR and SS among STEMI, NSTEMI, and UA patients. There was a statistically significant difference found between increased age and high SS also between low LVEF with high SS. NLR was also not associated with severity of CAD. Further studies with a larger sample size and long-term follow-up for outcomes are needed to conclusively evaluate the role of UAR in this group of patients.
